# In Vitro Investigation of Gelatin/Polycaprolactone Nanofibers in Modulating Human Gingival Mesenchymal Stromal Cells

**DOI:** 10.3390/ma16247508

**Published:** 2023-12-05

**Authors:** Zhiwei Tian, Zhongqi Zhao, Marco Aoqi Rausch, Christian Behm, Hassan Ali Shokoohi-Tabrizi, Oleh Andrukhov, Xiaohui Rausch-Fan

**Affiliations:** 1 Competence Center for Periodontal Research, University Clinic of Dentistry, Medical University of Vienna, 1090 Wien, Austria; n11945430@students.meduniwien.ac.at (Z.T.); zhaozhongqi0821@outlook.com (Z.Z.); marco.rausch@meduniwien.ac.at (M.A.R.); christian.behm@meduniwien.ac.at (C.B.); 2 Clinical Division of Orthodontics, University Clinic of Dentistry, Medical University of Vienna, 1090 Wien, Austria; 3 Core Facility Applied Physics, Laser and CAD/CAM Technology, University Clinic of Dentistry, Medical University of Vienna, 1090 Wien, Austria; hassan.shokoohi-tabrizi@meduniwien.ac.at; 4 Center for Clinical Research, University Clinic of Dentistry, Medical University of Vienna, 1090 Wien, Austria; xiaohui.rausch-fan@meduniwien.ac.at; 5 Division of Conservative Dentistry and Periodontology, University Clinic of Dentistry, Medical University of Vienna, 1090 Wien, Austria

**Keywords:** human gingival mesenchymal stromal cells, gelatin, polycaprolactone, collagen, electrospinning, periodontal tissue

## Abstract

The aesthetic constancy and functional stability of periodontium largely depend on the presence of healthy mucogingival tissue. Soft tissue management is crucial to the success of periodontal surgery. Recently, synthetic substitute materials have been proposed to be used for soft tissue augmentation, but the tissue compatibility of these materials needs to be further investigated. This study aims to assess the in vitro responses of human gingival mesenchymal stromal cells (hG-MSCs) cultured on a Gelatin/Polycaprolactone prototype (GPP) and volume-stable collagen matrix (VSCM). hG-MSCs were cultured onto the GPP, VSCM, or plastic for 3, 7, and 14 days. The proliferation and/or viability were measured by cell counting kit-8 assay and resazurin-based toxicity assay. Cell morphology and adhesion were evaluated by microscopy. The gene expression of collagen type I, alpha1 (COL1A1), α-smooth muscle actin (α-SMA), fibroblast growth factor (FGF-2), vascular endothelial growth factor A (VEGF-A), transforming growth factor beta-1 (TGF-β1), focal adhesion kinase (FAK), integrin beta-1 (ITG-β1), and interleukin 8 (IL-8) was investigated by RT-qPCR. The levels of VEGF-A, TGF-β1, and IL-8 proteins in conditioned media were tested by ELISA. GPP improved both cell proliferation and viability compared to VSCM. The cells grown on GPP exhibited a distinct morphology and attachment performance. COL1A1, α-SMA, VEGF-A, FGF-2, and FAK were positively modulated in hG-MSCs on GPP at different investigation times. GPP increased the gene expression of TGF-β1 but had no effect on protein production. The level of ITG-β1 had no significant changes in cells seeded on GPP at 7 days. At 3 days, notable differences in VEGF-A, TGF-β1, and α-SMA expression levels were observed between cells seeded on GPP and those on VSCM. Meanwhile, GPP showed higher COL1A1 expression compared to VSCM after 14 days, whereas VSCM demonstrated a more significant upregulation in the production of IL-8. Taken together, our data suggest that GPP electrospun nanofibers have great potential as substitutes for soft tissue regeneration in successful periodontal surgery.

## 1. Introduction

The presence of mucogingival deformities such as gingival recession does not merely affect the function of dentition but also influences the esthetics of patients. The structure deficiencies of gingiva can be caused by numerous etiological factors, including periodontal inflammatory infections, abnormal mechanical forces such as vigorous tooth brushing, occlusal trauma, orthodontic tooth movement, suboptimal dentures, insufficient keratinized tissue, a thin phenotype of gingiva, a buccal malposition of teeth, and an aberrant pull of labial frenulum [1,2,3,4]. In many cases, the lesion is a comprehensive outcome of multi-factors rather than a single stimulus [2,4]. Among all the soft tissue deficiencies, gingival recession and insufficient keratinized tissue are the most prevalent ailments [5]. The interest in mucogingival surgery, which is conducted to reconstruct gingival tissue defects, has gained popularity among clinicians and researchers [6].

The ultimate goal of periodontal mucosal therapy is to correct tissue defects, recover the original structure of periodontium, and improve the esthetical tissue profile [7]. Since the conception of mucogingival surgery was introduced by Friedman et al. [8], an increasing number of surgical procedures and tissue grafts have been established [7]. The xenogeneic collagen matrix is considered as a feasible material in the treatment of mucogingival defects [9,10]. After undergoing cross-linking for reinforcement, reconstituted porcine-derived collagen is transformed into a new porcine-derived VSCM, commercially known as Fibro-Gide^®^, and has recently been introduced for soft tissue regeneration. In vitro and animal studies have demonstrated favorable biocompatibility and the ability of tissue reconstruction using VSCM [10,11]. In addition, clinical studies have shown promising outcomes in terms of acquiring soft tissue volume and being comparable with subepithelial connective tissue grafts [12]. However, some researchers have argued that the xenogeneic collagen matrix hardly achieves clinical predictability and superiority in tissue volume augmentation [9,13]. Particularly, collagen-based materials tend to degrade rapidly and have poor biostability and mechanical properties, restricting the clinical application scenarios [14]. Meanwhile, the laborious production process and relatively high cost also hinder its clinical use [15]. In this sense, there are still no ultimate graft materials for periodontal soft tissue reconstruction.

Nano/microfiber materials have gained widespread application in recent years due to their ability to emulate the properties of the extracellular matrix (ECM) [16,17]. Electrospinning, which employs natural proteins or synthetic polymers, serves as a potent technique for creating scaffolds replicating the nanostructures inherent to the biological ECM [1,18]. Electrospun polycaprolactone (PCL) has gained popularity in the application of dental repair [1,19] and has been proven to support the proliferation of various cell types [20,21,22]. Furthermore, clinical research has corroborated the efficacy of PCL in facilitating tissue regeneration [23]. Despite the excellent mechanical properties of PCL, the pronounced hydrophobicity and lack of a biological recognition signal restrict certain clinical applications [24]. To compensate for these shortcomings, PCL is often used in conjunction with natural polymers, such as gelatin (GE). GE investigated for application in tissue regeneration is obtained by partially hydrolyzing the natural collagen [25,26]. The small size of cross-linking GE fibers is capable of mimicking the ECM, which structurally and biochemically supplies nearby cells [25]. Sarker et al. stated that high GE content integrated with the three-dimensional composite structure could effectually support the osteogenic differentiation of adipose-derived stem cells [27]. The electrospun fibers produced from a blend of GE and PCL have been proven in various tissue engineering studies to exhibit favorable mechanical strength and biocompatibility [16,28]. However, due to the distinct physicochemical properties of PCL and GE, their blend tends to undergo phase separation, compromising the electrospinning process and forming inhomogeneous structures with suboptimal blending [29]. Notably, a 3D sandwich-structured prototype (GPP), which integrates PCL electrospun fibers with GE electrospun nanofibers using a lamination technique, has been demonstrated to be a promising material. The PCL layer enhances the mechanical properties of the material without compromising its biocompatibility, while on the other hand, the surface GE layer achieves a degree of water resistance through in-situ crosslinking and retains the hydrophilicity of nanofibers [17,30].

The ideal bio-substitute for periodontal surgery should have certain characteristics, including biocompatibility, biodegradability, proper malleability, simplifying surgical manipulation, and facilitating cell proliferation, adhesion, and differentiation [10,15,31]. The remarkable tissue regeneration ability of the gingiva renders it an attractive tissue for cell isolation, both for therapeutic applications and research purposes. hG-MSCs can be readily isolated from gingival tissue possessing a range of functions, encompassing the regulation of oral tissue homeostasis and regeneration, and the modulation of inflammatory responses [32,33,34]. In the above sense, exploring the response of hG-MSCs to GPP, VSCM, and TCP holds significant promise not only for applications in regenerative medicine, but also for the development of 3D cell culture models focusing on complex cell-matrix interactions.

A previous study from our research group has validated the integrity of the material’s specific structure along with its biocompatibility [35]. However, the potential of GPP for oral soft tissue regeneration has not been well investigated. Especially, the interaction with hG-MSCs, which are vital for gingival tissue regeneration, has yet to be studied. Our study aims to investigate the interaction of GPP with hG-MSCs in vitro and to compare the biological properties of GPP with those of the aforementioned VSCM.

## 2. Materials and Methods

### 2.1. Ethics

The study was conducted in compliance with the Declaration of Helsinki regarding the ethical principles for medical research involving human subjects. The protocol for the isolation and work with hG-MSCs was approved by the Ethics Committee of the Medical University of Vienna, Vienna, Austria (vote no. 1079/2019, extended in 2023). Patients gave their informed and written consent before tooth donations.

### 2.2. Cell Isolation

Primary hG-MSCs were isolated from the non-inflamed and non-carious third molars extracted due to orthodontic indications from five periodontally healthy patients, as previously described [32]. Gingival tissue was removed from the tooth using a scalpel. The tissue pieces were cultured in Petri dishes using Dulbecco’s modified Eagles Medium (DMEM, Sigma-Aldrich, St. Louis, MO, USA), supplemented with 10% fetal bovine serum (FBS, Gibco, Carlsbad, CA, USA), 100 U/mL of penicillin, and 50 µg/mL of streptomycin (P/S, Gibco, Carlsbad, CA, USA). The dishes were kept at 37 °C and 5% CO_2_ under an atmosphere of 95% humidity. After outgrowing, the adherent cells were detached with Accutase^®^ (Sigma-Aldrich, St. Louis, MO, USA) and expanded in culture flasks. The cells in passages 4–6 were used in the experiments. hG-MSCs from each donor were separately evaluated. hG-MSCs were verified according to the previous study [36]. Mesenchymal surface markers CD29, CD73, CD90, CD105, and CD146 were positively tested, and hematopoietic surface markers (CD31, CD34, and CD45) were negatively stained.

### 2.3. Preparation of GPP and VSCM

A GPP with diameters of fibers varying from 90 to 680 nm was fabricated by Neo Modulus [Suzhou] Medical (Suzhou, China). The Freiburg Materials Research Center and Institute for Macromolecular Chemistry of the Albert-Ludwigs University Freiburg supplied the technology used to manufacture GPP, and the detailed structure and production methodology have been described in previous studies [30,35]. The VSCM (Fibro-Gide^®^), comprising type I and type III porcine collagen with a porosity of 93% by volume and an average pore size of 92 μm [11], was purchased from Geistlich Pharma (Wolhusen, Switzerland). GPP was prepared by punching out 6 mm diameter cylindrical specimens utilizing a corneal trephine (Yanlijian Technologies, Shenzhen, China) under sterile conditions, and VSCM was first cut into 3 mm thick slices and then punched out 6 mm specimens, which were placed at the well bottoms of 96-well plates for the experiments. The representative samples and scanning electron microscopy images of both materials are provided in Supplementary Figure S1. Tissue culture plastic (TCP) served as a control according to the previous studies [31,35]. Scanning electron microscopy of GPP and VSCM was performed as described previously [35,37].

### 2.4. Cell Morphology and Adhesion

Cell adhesion was analyzed by imaging the morphology of adhered cells by staining the nuclei of hG-MSCs and actin cytoskeleton structures. Firstly, hG-MSCs were cultured on different materials with an initial seeding density of 5 × 10^3^/well in 200 μL of DMEM. After 3, 7, and 14 days, cells were fixed on the GPP, VSCM, and TCP surface by incubating with 4% paraformaldehyde (ThermoFisher Scientific, Waltham, MA, USA) for 15 min and afterwards permeabilized by treating with 0.1% Triton-X100 for 5 min at room temperature. After blocking by bathing in 1% bovine serum albumin for 30 min and triple washing with PBS, rhodamine-conjugated phalloidin (1:200, ThermoFisher Scientific, Waltham, MA, USA) was added. After incubation for 45 min and three washing steps, nucleus counterstain was performed with 4′, 6-diamidino-2-phenylindole (DAPI, 1:1000 in PBS, Sigma-Aldrich, St. Louis, MO, USA) and incubated for 5 min at room temperature. The cell morphology and adhesion were observed under a fluorescence microscope using the ECHO Revolve fluorescence microscope (Echo, San Diego, CA, USA).

### 2.5. Cell Proliferation/Viability and Metabolic Activity Assays

First, 200 μL of DMEM with 2.5 × 10^4^ hG-MSCs/mL was seeded onto GPP, VSCM, or TCP, respectively. Consequently, the total cell number on each surface was approximately 5 × 10^3^. The proliferation/viability was determined by a cell counting kit (CCK-8, Dojindo Laboratories, Kumamoto, Japan) on 3, 7, and 14 days. CCK-8 reagent was added at 10% and incubated for 4 h. The amount of formazan dehydrogenized from tetrazolium salt was measured photometrically at 450 nm wavelength using a microplate reader (Synergy HTX; BioTek, Winooski, VT, USA). Metabolic activity was tested using a resazurin-based toxicity assay (Sigma-Aldrich, St. Louis, MO, USA). Here, 20 μL of resazurin-based solution was added to culture wells containing 200 μL of cell solution, followed by incubation for 6 h. The reduction of resazurin to resorufin was monitored using a fluorescence-based plate reader (Synergy HTX; BioTek) at 540/34 nm excitation and 600/40 nm emission wavelength. The assays were performed with hG-MSCs isolated from 5 individual donors and two technical replicates.

### 2.6. RT-qPCR and ELISA

hG-MSCs were seeded onto each type of surface at an initial density of 3 × 10^4^/well in 200 μL of DMEM. After 3, 7, and 14 days of incubation, hG-MSCs were lysed and mRNA was transcribed into cDNA using the following TaqMan gene expression assays (Applied Biosystems, Foster City, CA, USA): COL1A1 (Hs00164004_m1), VEGF-A (Hs00900055_m1), TGF-β1 (Hs00998133_m1), ACTA2 (Hs00909449_m1), FAK (Hs00169444_m1), ITG-β1 (Hs01127536_m1), FGF-2 (Hs00266645_m1), IL-8 (Hs00174103_m1), and glyceraldehyde-3-phosphate dehydrogenase (GAPDH, Hs99999905_m1). Reverse transcription reaction was performed with the following conditions: 37 °C for 1 h and 95 °C for 5 min using Primus 96 advanced thermocycler (Peq/Lab/VWR, Darmstadt, Germany). Quantitative analysis of the gene expression was carried out by using a QuantStudio 3 device (Applied Biosystems, Foster City, CA, USA). All the samples were heated at 95 °C for 10 min and then followed with 50 cycles, with the denaturation of each process under 95 °C for 15 s and annealing/extension at 60 °C for 1 min. The expression results of the target gene were generated based on 2^−ΔΔCt^ calculations with a control sample based on a previous study [38]. GAPDH was used as a reference gene. hG-MSCs seeded on TCP and incubated for 3 days served as a control.

The levels of VEGF-A, TGF-β1, and IL-8 proteins in the conditioned media were determined after 3, 7, and 14 days of incubation using a human VEGF-A kit (Cat. Nr. BMS277-2, ThermoFisher Scientific, Waltham, MA, USA), a human TGF-β1 kit (Cat. Nr. CEK1332, Cohesion Biosciences, London, UK), and a human IL-8 ELISA kit (Cat. Nr. 88-8086, ThermoFisher Scientific, Waltham, MA, USA), respectively, according to the manufacturer’s protocols, and using a Synergy HTX multi-mode reader (BioTek Instruments, Winooski, VT, USA).

### 2.7. Statistical Analysis

All data were analyzed using GraphPad Prism software (version 9, La Jolla, CA, USA). Quantitative data were summarized in terms of the mean ± standard deviation (SD). Normal distribution was proved by the Kolmogorov–Smirnov test. For the parametric data, the differences between groups were assessed by one-way analysis of variance (ANOVA) for repeated measures. A non-parametric Friedman test was applied in case the data were not normally distributed. Differences were considered significant at *p* < 0.05.

## 3. Results

### 3.1. Cell Morphology and Adhesion

**Figure 1:**
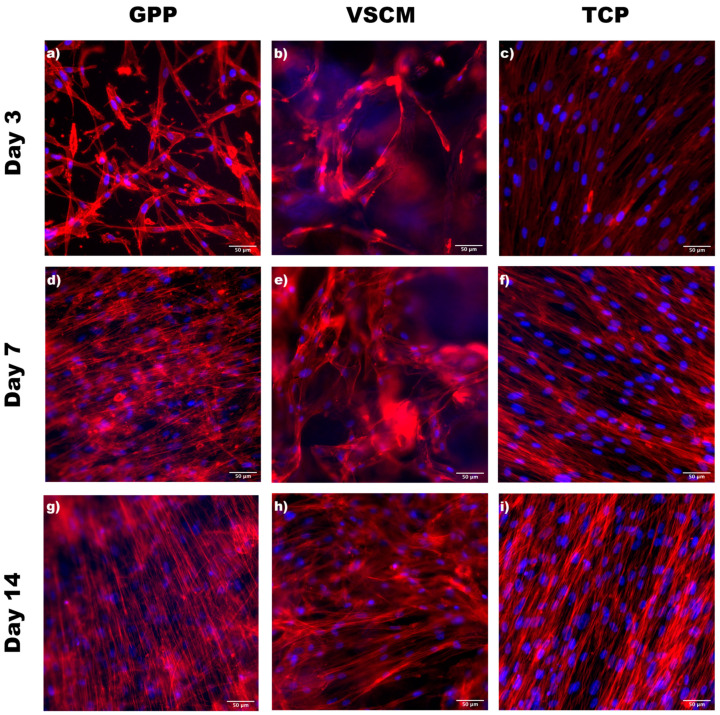
Morphology of hG-MSCs on different surfaces. Representative fluorescence microscopy images of hG-MSCs on GPP (**a**,**d**,**g**), VSCM (**b**,**e**,**h**), and TCP (**c**,**f**,**i**). Actin filaments (red) and nuclei (blue) of cells were stained after 3, 7, and 14 days. Images show one representative donor and were taken at 100-fold magnification. Scale bars correspond to 50 µm. It can be observed that the morphology of cells adhered to the three different materials varied.

To examine the adhesion and morphology of hG-MSCs cultured on different materials, they were stained with phalloidin (F-actin) and DAPI (nucleus), and images were taken using fluorescence microscopy (Figure 1). The hG-MSCs seeded over the TCP presented a typically elongated and flat morphology after 3 days of incubation, which could be regarded as an indicator for high-adhering cells. Despite VSCM showing a stained collagen matrix with red and blue color interfering with the foreground image of cells, pyramidal-shaped hG-MSCs attached to the materials were observed. Contrarily, the cells seeded on GPP exhibited a more elongated and flatter morphology than the cells on VSCM. Furthermore, the cells on GPP displayed several filopodia attached to the fiber, indicating that cells could migrate properly on the structure. After 7 and 14 days of incubation, the cells grown on GPP were completely spread and with relatively aligned actin filaments, whereas the cells on TCP and VSCM exhibited randomly oriented filaments. In addition, hG-MSCs seeded on VSCM had a mixed morphology with stretch-shaped and polygonal cells after 7 days; however, more stretched cells could be observed after 2 weeks.

### 3.2. Cell Proliferation/Viability and Metabolic Activity

**Figure 2:**
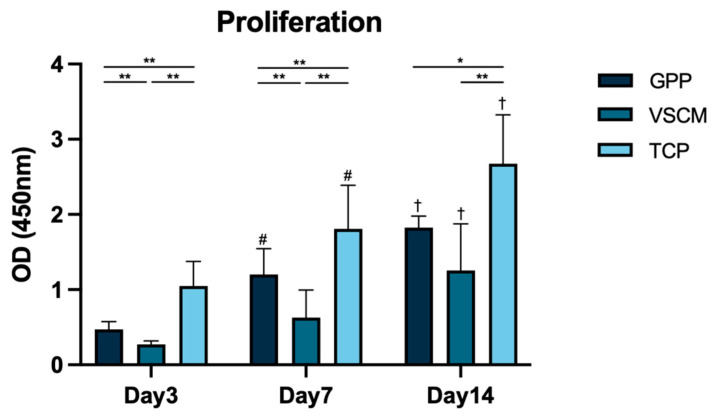
The proliferation/viability of hG-MSCs on GPP, VSCM, and TCP. The proliferation/viability of cells was measured with CCK-8 after 3, 7, and 14 days. Cells seeded on TCP served as control. The data are shown as the mean ± SD (*n* = 5). Differences between groups at the same time point are indicated by * (*p* < 0.05) and ** (*p* < 0.01), whereas those between days 3 and 7 and days 7 and 14 for the same material are indicated with # (*p* < 0.01) and † (*p* < 0.01), respectively. GPP is more effective than VSCM in facilitating the proliferation/viability of hG-MSCs.

The effect of different materials on the proliferation of hG-MSCs was investigated after 3, 7, and 14 days (Figure 2). The cells seeded on TCP possessed the highest proliferation level at all time points of measurement (*p* < 0.05), and the proliferation of hG-MSCs seeded on GPP was significantly higher compared with that on VSCM after 3 and 7 days of incubation (*p* < 0.01). On day 14, formazan dye also tended to be higher in the hG-MSCs seeded on GPP than in the cells grown on VSCM (*p* = 0.07). Cell growth on GPP and TCP was promoted over time, whereas hG-MSCs seeded on VSCM maintained a relatively slow proliferation rate during the early incubation time.

**Figure 3:**
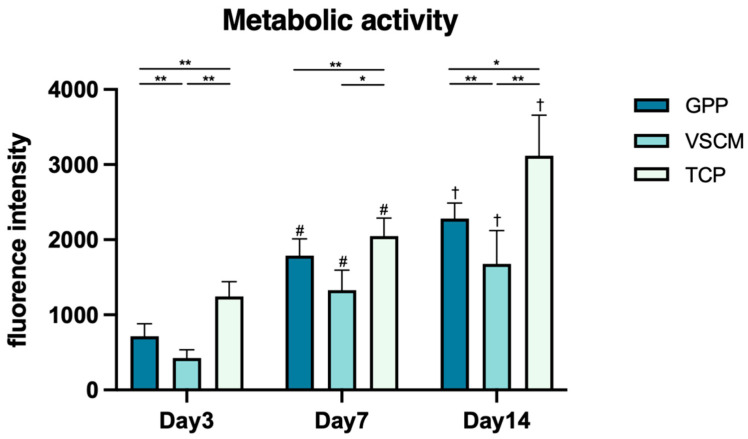
The effects of GPP, VSCM, and TCP on the metabolic activity of hG-MSCs. The amount of metabolized resazurin produced by cells was measured after 3, 7, and 14 days. Cells seeded on TCP served as control. The data from five individual donors are represented as the mean ± SD. * and **—significantly different between cells seeded on different surfaces of materials at the same time point of measurement with *p* < 0.05 and *p* < 0.01, while the distinctions between days 3 and 7 and days 7 and 14 for the same material are indicated by # (*p* < 0.01) and † (*p* < 0.05), respectively. Compared to VSCM, GPP promoted the metabolic activity of hG-MSCs.

hG-MSCs were metabolically active on different material surfaces (Figure 3). Consistent with the proliferation, the resorufin production level was the highest in cells seeded on TCP (*p* < 0.05). hG-MSCs grown on GPP had significantly higher metabolic activity than those grown on VSCM after 3 and 14 days of incubation (*p* < 0.01). Nonetheless, a similar tendency was observed after 7 days; the differences were not statistically significant (*p* = 0.07). The metabolic activities of cells growing on all the different substrates continuously increased within 2 weeks.

### 3.3. Effects of GPP and VSCM on Gene Expression

**Figure 4:**
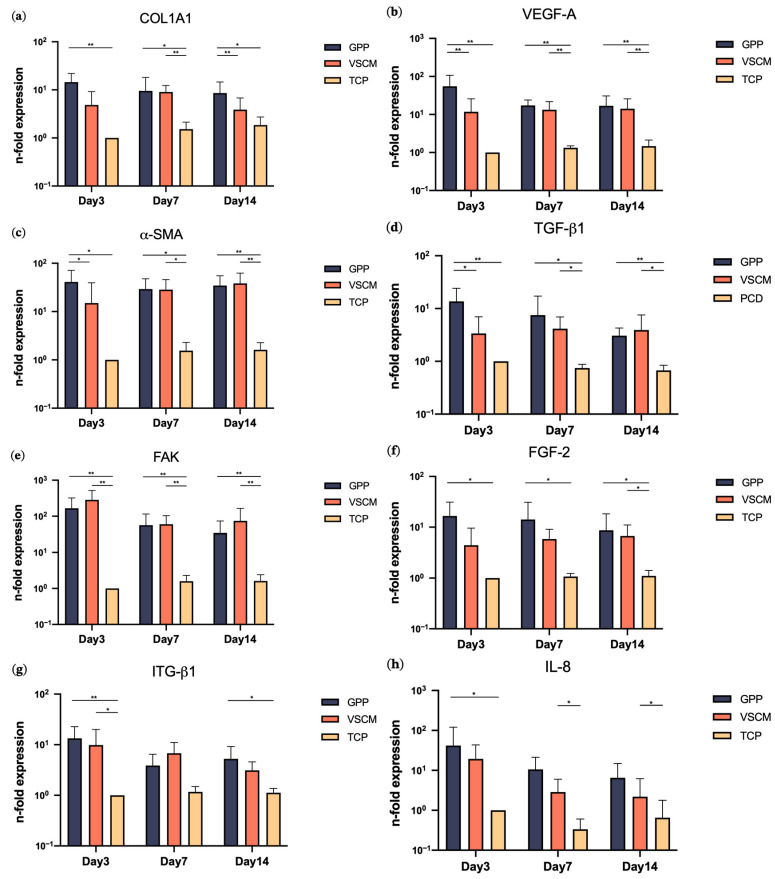
Gene expression of various proteins in hG-MSCs grown on different materials. Gene expression levels of COL1A1 (**a**), VEGF-A (**b**), ACAT2 (**c**), TGF-β1 (**d**), FAK (**e**), FGF-2 (**f**), ITG-β1 (**g**), and IL-8 (**h**) were assessed by RT-qPCR after 3, 7, and 14 days of culture, calculated using the 2^−ΔΔCt^ method and GAPDH as a reference gene. Y-axes show the n-fold expression levels compared to the level of day 3 of TCP control (n-fold expression = 1). The data are displayed as the mean ± SD of five different donors. Differences between groups at the same time point are indicated by * and ** for *p* < 0.05 and *p* < 0.01, respectively. hG-MSCs cultured on biomaterials exhibited increased gene expression of the investigated biomarkers compared to those on TCP. Cells cultured on GPP demonstrated slightly yet significantly increased expression of VEGF-A, α-SMA, and TGF-β1 after 3 days, and COL1A1 after 14 days, compared to those grown on VSCM.

Figure 4 shows the expression level of different molecular makers in hG-MSCs cultivated on GPP, VSCM, and TCP. In all cases, the gene expression of all investigated proteins was higher in the cells growing on the biomaterials compared to TCP, and in many cases, these differences were statistically significant. The differences between different materials were rather marginal; hG-MSCs growing on GPP showed minorly but significantly higher expression of VEGF-A, α-SMA, and TGF-β1 after 3 days and COL1A1 after 14 days of culture compared to VSCM.

### 3.4. Effects of GPP and VSCM on the Protein Production by hG-MSCs

**Figure 5:**
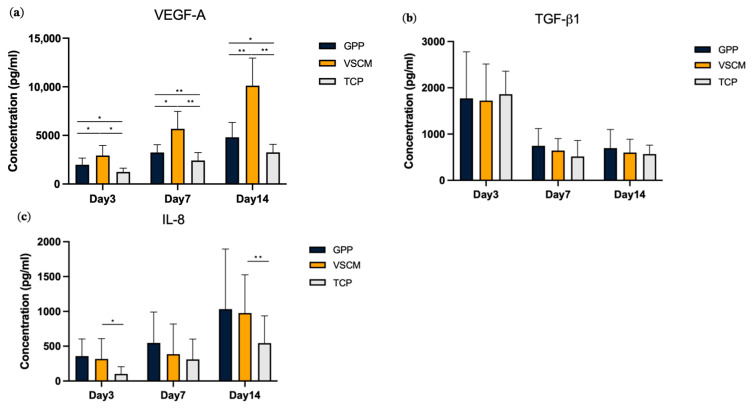
Protein production by hG-MSCs grown on different materials. The protein production of VEGF-A (**a**), TGF-β1 (**b**), and IL-8 (**c**) in the cells cultured on different materials for 3, 7, and 14 days was quantified by ELISA. Y-axes show the concentration of target protein in conditioned media. The data from five donors are displayed as the mean ± SD. Differences between groups at the same time point are indicated by * and ** for *p* < 0.05 and *p* < 0.01, respectively. Cells grown on VSCM presented the highest expression level of VEGF-A. VSCM showed a more significant upregulation in the production of IL-8 after 3 and 14 days. There was no significant difference in the production of TGF-β1 among the three groups.

Figure 5 represents the protein production level of VEGF-A, TGF-β1, and I-L8 in different culture conditions. The production of VEGF-A was found to be highest in cells growing on VSCM compared to GPP and TCP at all time points (Figure 5a). Furthermore, higher production of VEGF-A was observed for hG-MSCs growing on GPP compared to those growing on TCP. No difference in the content of TGF-β1 in the conditioned media was observed between the surfaces at all time points (Figure 5b). Slightly higher levels of IL-8 were observed in cells growing on biomaterials compared to TCP, but significant differences were observed only between VSCM and TCP after 3 and 14 days of culture (Figure 5c).

## 4. Discussion

The execution of the physiological function of various cell types is regulated not only by the biological signals, but also by the ECM and the surrounding cells [39]. A wide array of scaffold designs share a common objective: constructing an ideal structure that resembles the ECM of native tissues. The electrospinning process represents a straightforward, efficient, versatile, cost-effective approach that is capable of generating nanometer-scale non-woven fibrous structures, making it particularly suitable for applications such as wound dressing and tissue engineering [16,40]. A three-dimensional (3D) scaffold, when constructed from nanofibers, is expected to yield a biomimetic structure that is akin to the ECM [41].

Cell attachment to materials serves as a fundamental precursor to various other cellular phenomena, including proliferation, spreading, migration, and differentiation. In this study, hG-MSCs exhibited recognition and attachment to three different materials. After 3 days of cell seeding, cells demonstrated enhanced and accelerated spreading on GPP in comparison to the collagen matrix; meanwhile, the formation of filopodia by cells growing on the surface of GPP further confirmed the presence of a substantial contractile force facilitating the cell migration (Figure 4). Consistent with the current data, Jiang et al. substantiated that nanofiber materials comprising different PCL/GE ratios displayed desirable morphology and adhesion of human bone marrow-derived MSCs [42]. In contrast to VSCM, GPP manifested a notable occurrence of cells joining together and/or overlapping. This observation indicated that the cells were engaging in interactions with one another. However, a prior study demonstrated some disparities, revealing that collagen nanofiber exhibited notably better cytocompatibility with mouse fibroblasts when compared to GE nanofiber [43]. One potential reason could be that electrospun GPP possesses a higher density of Arg-Gly-Asp (RGD) cell-binding sites compared to reconstituted VSCM [11,17,30]. In addition, nanometer-scale fibers could be more conducive to promoting cell attachment and extension than micrometer-scale materials [44]. This may provide another explanation for why nanoscale GPP presented different cell adhesion to micro-scale VSCM.

Cell proliferation and viability are also pivotal factors to consider when determining the biocompatibility and suitability of a scaffold for tissue regeneration. The CCK8 test was conducted to assess the proliferation/viability of hG-MSCs on different substrates. Although GPP exhibited inferior capabilities compared to the positive control group, the number of cells growing on it increased continuously over 2 weeks (Figure 2). These outcomes are coherent with numerous previous studies, which indicated that fibroblasts, osteoblasts, and cardiac muscle cells all exhibited favorable non-toxicity and high proliferation rates on PCL/GE electrospinning scaffolds [29,41,45]. Curiously, the proliferation of cells seeded on VSCM appeared to remain unchanged during the first week, indicating the delayed growth of hG-MSCs on this substrate. The data on the metabolic activity of the cells generally affirmed this conclusion.

Collagen is essential for preserving the biological and structural completeness of the ECM configuration. The interaction between cells and the collagen secreted by themselves is crucially implicated in cell adhesion and spreading, thereby playing a significant role in determining cellular differentiation pathways [46]. Furthermore, collagen type I is the primary constituent found in periodontal connective tissue. The cultivation of hMSCs on PCL/GE hybrid nanofiber materials has been shown to effectively promote the expression of COL1A1, which is in parallel with our study that GPP manifested an upregulation of COL1A1 until 14 days [47]. Surprisingly, VSCM enhanced the gene expression of COL1A1 less effectively than GPP. In contrast to COL1A1, the expression of FAK, which activates multiple signal transduction pathways that ultimately lead to promoted proliferation and migration, demonstrated a significant increase by GPP and VSCM in 2 weeks, respectively. Prior research has indicated consistent findings that both the GE scaffold and PCL/collagen nanofibers play crucial roles in the production of FAK [48,49]. Simultaneously, cells engage with the ECM via integrin molecules, notably ITG-β1, leading to the activation of FAK [50]. The data we obtained revealed a notable upsurge in the expression of ITG-β1 within the GPP and VSCM groups compared to the TCP group after 3 days, whereas GPP induced a higher expression of this protein after 2 weeks. A similar finding was reported by a previous study showing a higher expression of ITG-β1 in human MSCs in a PCL-GE nanofiber environment than TCP after 21 days [51]. Further, actin is a prominent cytoskeletal protein that plays a vital role in facilitating cellular locomotion [52]. A specific PCL-GE electrospun material exhibits an increase in the expression of α-SMA in human smooth muscle cells [53]. In the same manner, α-SMA experienced upregulation due to GPP within 14 days, whilst VSCM appeared to exert a positive effect after the first week. Combining the above findings, it can be inferred that both GPP and VSCM positively regulate ECM generation and cellular functionality activation. Moreover, GPP demonstrated a slightly superior effect in comparison to VSCM.

Angiogenesis, the process of forming novel blood vessels, holds significant importance in numerous biological processes, with particular significance in the context of wound healing [54]. VEGF is a potent growth factor known for its ability to stimulate the formation of blood vessels through angiogenesis [55]. TGF-β1 is also involved in angiogenesis and serves as a pivotal immunosuppressive secreted polypeptide. Moreover, as a versatile growth factor, it governs various behaviors and functions of fibroblasts and MSCs, thereby orchestrating tissue remodeling by producing connective tissue constituent and matrix proteins [56]. In our study, both VEGF-A and TGF-β1 mRNA levels increased when cultured on GPP within a two-week period, whereas VSCM only showed significant effects after 7 days. Similar to our experiment, several studies have also shown that PCL, GE, and collagen composite structures could promote the production of VEGF and TGF-β1 [57,58,59]. However, in terms of the protein content of VEGF-A in the conditioned media, VSCM demonstrated the highest values among the three groups. Additionally, regarding the concentration values of the TGF-β1 protein, the levels remained consistent across all materials. The discrepancy between the levels of gene expression and the subsequent quantity of protein expression could be attributed to various factors. First, gene expression is measured at a single time point, whereas the protein content reflects the total protein production for a certain time period. Moreover, distinct materials and specific proteins may interact in a different way. Collagen and GE structures have both been proven as effective carriers for the controlled release of growth factors [55,60]. It is mentionable that nanoscale structures are known for their capacity to provide a larger surface area for protein absorption [61]. Moreover, endogenous multifunctional FGF-2, which has been demonstrated to enhance human MSC proliferation and mediate the interaction of MSCs with the ECM matrices, sustains high expression in hG-MSCs on GPP [62]. It was only after 14 days that VSCM showed differences compared to the control. It could be observed that both GPP and VSCM shared comparable positive regulatory functions in angiogenesis and ECM remodeling, with a slightly superior effect of GPP.

The utilization of grafts and substitute materials has been restricted because of the foreign body reaction, which constitutes an initial acute sterile inflammatory response [63]. Consequently, the extended overexpression of pro-inflammatory factors, such as IL-8, IL-6, and others, can result in an excessive inflammatory response, ultimately compromising the integrity of the surrounding tissue [32]. According to the current findings, it seemed that both GPP and VSCM consistently upregulated the gene expression of IL8 throughout the entire duration. At the protein production level, qualitatively similar data were obtained. Gil-Castell et al. obtained correlated results where the PCL/GE nanofiber induced the expression of pro-inflammatory factors IL6 and IL1b after 48 and 72 h. However, histological examination results revealed that there was no noticeable inflammation after two weeks [45]. It appears that both GPP and VSCM, through the prolonged overexpression of IL8, may potentially lead to a slightly higher inflammatory response. Nonetheless, further comprehensive and in-depth explorations in this field are required for a thorough understanding.

Biomaterials primarily consisting of collagen or GE often bring out similar outcomes due to the close similarity in their biochemical properties [26,64]. However, in this experiment, GPP and VSCM had slightly different effects on the specific behavior of hG-MSCs. Apart from the intrinsic biochemical cues within the structure, surface topology features also have a significant impact on cellular behavior [65]. The orientation of scaffold fibers can impact not only the morphology and arrangement of cells, but also potentially induce alterations in gene expression [66]. These topographical cues could potentially influence the reorganization of the cell nucleus, leading to alterations in gene expression [67]. The unique surface topography resulting from the diameter and arrangement of fibers in GPP and VSCM leads to cell morphology and distribution variations, eventually resulting in potential variations in cellular functional behaviors, including gene expression [35,68]. The mechanical properties of the fiber structure itself are often a key determinant in influencing cellular morphology and arrangement. Previous research has indicated that increasing GE content does not necessarily lead to the improved proliferation and adhesion of MSCs. Instead, the most favorable cellular responses were obtained when the ratio of PCL to GE was 2:1 [69]. In addition to enhancing material mechanical strength and stability through cross-linking, like VSCM, the PCL layer in GPP further reinforces its stiffness. This not only enables the versatile application of GPP in vivo, but also holds significant potential for modifying the behavior of the cells settled on it.

The main limitation of this experiment lies in its inability to mimic the real 3D in vivo environment accurately. The absence of biochemical signaling networks and physical conditions like fluid dynamics may render the final results relatively biased. Furthermore, the lack of more comprehensive and in-depth research on the interplay between physicochemical properties and cellular behavior hinders materials from accurately simulating the ECM environment. To address these restrictions, future research should encompass more extensive studies regarding the correlation between alterations in material properties and the resulting changes in cellular activities, as well as their effectiveness in in vivo settings.

## 5. Conclusions

In summary, we have established an in vitro 3D model to evaluate the proliferation, viability, morphology, attachment, and gene expression of hG-MSCs within both GPP and VSCM. GPP promoted cell proliferation and viability compared to the collagen matrix. The cells cultured on GPP exhibited favorable morphology and a more organized arrangement. The cells cultured within both GPP and VSCM demonstrated an upregulation of genes linked to ECM generation, remodeling, the facilitation of ECM-cell interactions, and angiogenesis, with GPP displaying a relatively superior effect and a lower tendency in pro-inflammatory factor expression. These findings strongly support the suitability of GPP for applications in periodontal soft tissue regeneration procedures. Further physicochemical properties of GPP and in vivo investigations are required.

## Data Availability

The data presented in this study are available upon request from the corresponding author.

## Supplementary Materials

The following supporting information can be downloaded at: https://www.mdpi.com/article/10.3390/ma16247508/s1, Figure S1: Appearance of GPP and VSCM after preparation and their structure by SEM.
