# Supplementary material for: In Vitro Investigation of Gelatin/Polycaprolactone Nanofibers in Modulating Human Gingival Mesenchymal Stromal Cells
Source: Materials (Basel). 2023 Dec 5;16(24):7508. doi: 10.3390/ma16247508 (PMC10744501; doi:10.3390/ma16247508)
Supplement: Supplementary file 1 [file materials-16-07508-s001.zip › materials-2695517-supplementary.pdf]

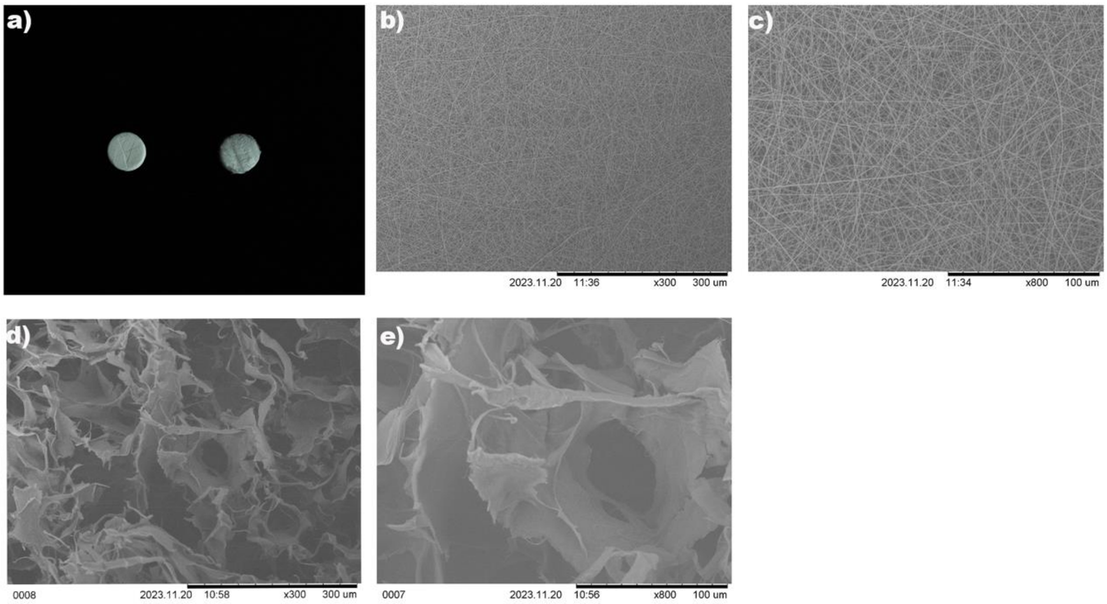

Figure S1. Appearance of GPP and VSCM after preparation and their structure by SEM. The punched discs are about 6 mm in diameter (a). Fiber structure of GPP under Scanning electron microscopy (b, c), Fiber structure of VSCM under Scanning electron microscopy (d, e).
